# Extensor-tendons reconstruction using autogenous palmaris longus tendon grafting for rheumatoid arthritis patients

**DOI:** 10.1186/1749-799X-3-16

**Published:** 2008-04-24

**Authors:** Po-Jung Chu, Hung-Maan Lee, Yao-Tung Hou, Sheng-Tsai Hung, Jung-Kuei Chen, Jui-Tien Shih

**Affiliations:** 1 Department of Orthopaedic Surgery, Taoyuan Armed Forces General Hospital, 168, Jong-Shing Rd, Taoyuan County, Taiwan

## Abstract

**Background:**

The purpose of the study is to retrospectively review the clinical outcome of our study population of middle-aged RA patients who had suffered extensor-tendon rupture. We reported the outcome of autogenous palmaris tendon grafting of multiple extensor tendons at wrist level in 14 middle-aged rheumatoid patients.

**Methods:**

Between Feb. 2000 to Feb. 2004, thirty-six ruptured wrist level extensor tendons were reconstructed in fourteen rheumatoid patients (11 women and three men) using autogenous palmaris longus tendon as a free interposition graft. In each case, the evaluation was based on both subjective and objective criteria, including the range of MCP joint flexion after surgery, the extension lag at the metacarpophalangeal joint before and after surgery, and the ability of the patient to work.

**Results and Discussion:**

The average of follow-up was 54.1 months (range, 40 to 72 months). The average range of MCP joint flexion after reconstruction was 66°. The extension lag at the metacarpophalangeal joint significantly improved from a preoperative mean of 38° (range, 25°–60°) to a postoperative mean of 16° (range, 0°–30°). Subjectively all patients were satisfied with the clinical results, and achieved a return to their level of ability before tendon rupture. We found good functional results in our series of interposition grafting using palmaris longus to reconstruct extensor tendon defects in the rheumatoid patients.

**Conclusion:**

Reconstruction for multiple tendon ruptures is a salvage procedure that is often associated with extensor lag and impairment of overall function. Early aggressive treatment of extensor tendon reconstruction using autogenous palmaris longus tendon as a free interposition graft in the rheumatoid wrist is another viable option to achieve good clinical functional result.

## Background

Wrist involvement is common for patients afflicted with rheumatoid arthritis (RA), wrist level tendon rupture commonly occurring in the extensor tendons of the ring and small fingers. Once a tendon rupture at wrist level has occurred, surgical techniques such as traditional end-to-side tendon transfer (suturing the distal portion of the ruptured tendon to an intact neighbouring tendon) or tendon repair with end-to-end interposition grafting can provide acceptable restoration of finger extensor function subsequent to such procedures. The purpose of the study is to retrospectively review the clinical outcome of our study population of 14 middle-aged RA patients who had suffered extensor-tendon rupture, and undergone tendon reconstruction incorporating autogenous palmaris tendon grafting of the multiple extensor tendon(s) at wrist level.

### Patients and methods

Thirty-six ruptured extensor tendons derived from fourteen RA patients (11 women and three men) were reconstructed during the period Feb. 2000 to Feb. 2004 inclusively. (Table 1) The mean age of study participants at time of surgery was 47.3 years (range, 32–66 years) and their mean of time lag between tendon rupture and surgery was 9.4 weeks. (range, 2 to 24 weeks) All of the involved patients have received some level of medical treatment for their arthritic condition. No patient had undergone any previous surgical treatment to their hand. Larsen's x-ray classification [1] was used to assess the relative severity of the rheumatoid arthritis from which each study participant suffered. In each case, we reconstructed extensor tendons using a section of autogenous palmaris longus tendon as a free interpositional tendon graft. The presence of this tendon was determined before grafting procedure at our outpatient department. Fourteen consecutive patients were operated on by one surgeon (J.T. Shih).

**Table 1:** General Data of Patients

Case	Gender	Age	Occupation	Involved Wrist*	Time lag(weeks) of tendon rupture to surgery	Tendon rupture	Advanced procedure
1	F	32	office lady	(R)	6	EDC4/EDM	
2	F	44	housewife	(R)	13	EDC4/EDC5/EDM	
3	F	46	nurse	L	7	EDC4/EDC5/EDM	Darrach procedure
4	F	52	baby-sitter	L	14	EDC2/EDC3	
5	M	40	soldier	R	5	EDC3/EDC5	
6	F	46	secretary	L	2	EDC4/EDC5/EDM	
7	F	54	housewife	(R)	12	EDC4/EDC5/EDM	
8	F	66	housewife	(R)	24	EDC4/EDC5	Sauve-Kapandji procedure
9	F	38	dustmen	L	8	EDC3/EDC4/EDM	
10	F	42	secretary	R	7	EDC2/EDC3/EDC4	
11	M	53	factory worker	(L)	12	EDC4/EDC5/EDM	
12	F	56	dinner lady	(R)	10	EDC4/EDC5/EDM	Darrach procedure
13	F	46	hairdresser	(R)	7	EDC5/EDM	
14	M	47	machine operatore	(R)	5	EDC4/EDC5	

*Parentheses indicate that the injury involved the dominant limb; EDC: extensor digitorum communis; EDM: extensor digitorum minimi

### Surgical Procedure

A dorsal incision was made in the mid-line extending from 5 cm proximally to 5 cm distally over the wrist, followed by the raise of the skin flaps. For the next step, the extensor retinaculum divided between the 5^th ^and the 6^th ^compartments, and reflected radially. Following this, the extensor tendons were exposed, and the synovium over the free ends of the tendons removed using a synovial rongeur. A dorsal synovectomy of the wrist was then completed, and the dorsal bony surfaces inspected for any bone spicules or abrasive irregularities that may have been present, all of which were subsequently removed. Next, the ruptured extensor tendons were identified, and mobilized as was deemed to be necessary, and the tendon ends were debrided and the defect in the length of the extensor tendon was estimated.

**Figure 1:**
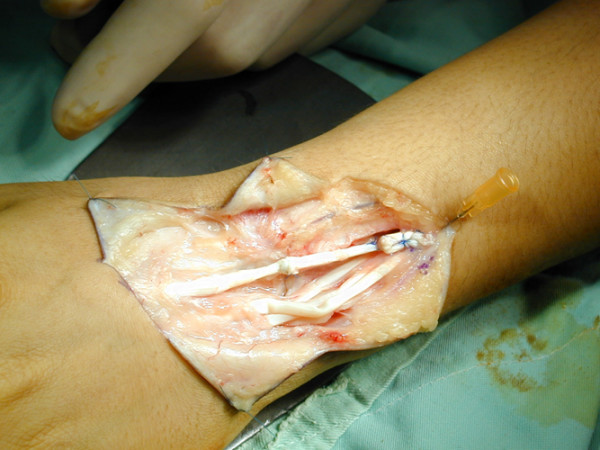
The extensor digitorum (ED) was reconstructed using free tendon grafting by means of a Pulvertaft technique.

Palmaris longus tendon was isolated by a transverse incision at wrist joint and then harvested using a tendon stripper or harvested through a number of small separate flexor incisions. Appropriate lengths of palmaris longus tendon were then used as free grafts to reconstitute the ruptured extensor tendons. The distal end of the damaged tendon was then secured by a series of six-strand sutures method with 4-0 prolene which was placed through the tendon graft and each of the ruptured tendon ends. (Fig. 1) Tensioning of the sutures was appropriately adjusted so that the graft was snug with the wrist in a position of 40° extension and with the metacarpophalangeal (MCP) joints in a 15° of flexion. Following this weaving of the proximal tendon stump was conducted using a Pulvertaft weave [2] secured with six-strand suture per anastomosis, and the reconstructed tendons were passed under the retinaculum. The repair was made in a position of 30° of wrist extension with the expectation that the repair wound stretch slightly as the wrist was exercised. Following this, the surgical wound was then closed.

At the time of tendon reconstruction, synovectomy of the distal radio-ulnar joint was also completed using a Darrach procedure for two cases in which the distal ulna was placed in a position of dorsal subluxation. Arthrodesis of the DRUJ (Sauve-Kapandji procedure) was conducted for one patient's wrist.

### Post-operative Management

Postoperatively, the hand was immobilized in a splint, with the wrist in 30 degrees of extension position in order to relieve stress at the repair site, and to maintain the metacarpophalangeal and interphalangeal joints in a neutral position. Passive interphalangeal-joint exercises were initiated 24–48 hours postoperatively and light active mobilization of the metacarpophalangeal and the involved wrist joint was commenced at two weeks post-surgery. The use of dynamic extensor tendon splintage for early mobilization of four weeks. Patients were weaned from the splint over a period of three days. On removal of the splint, active range of motion and tendon gliding exercises were started.

The average of follow-up was 54.1 months (range, 40 to 72 months). The latest evaluation was based on both subjective and objective criteria, including the range of MCP joint flexion after surgery, the extension lag at the metacarpophalangeal joint before and after surgery, and the ability of the patient to work. The active range of motion of all the study-participants' metacarpophalangeal joints flexion were measured subsequent to surgery. In each case, the extension lag, as determined at the metacarpophalangeal joint, was assessed both prior to and following tendon-repair surgery with a goniometer. Pearson's correlation coefficient was used to assess the correlation between the two quantitative variables. The ability to work was evaluated on the basis of whether the patient had returned to his or her original occupation and was able to work full-time (100 percent) or part-time (25, 50, or 75 percent of the normal time).

## Results

### Clinical Outcome

**Figure 2:**
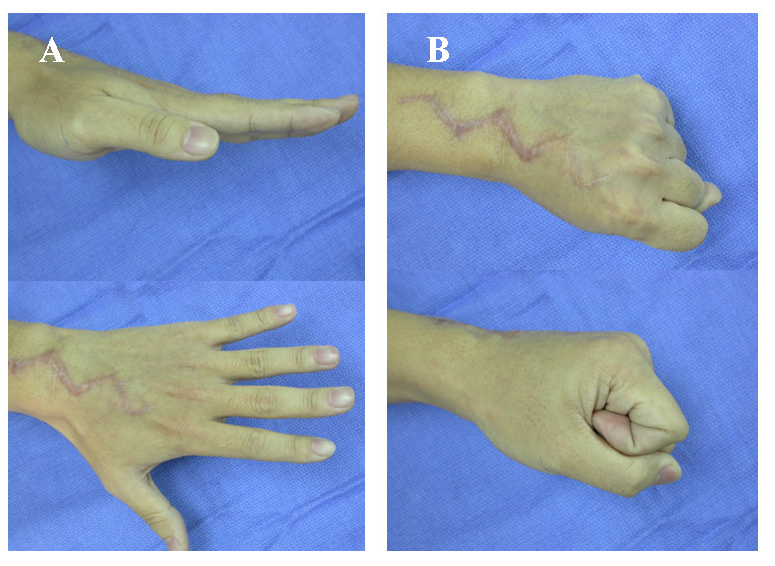
**Photograph of the wrist made at the latest follow-up evaluation.** Reconstruction of a ruptured extensor tendon conducted by means of free-tendon grafting. A, Extension and B, Flexion.

Fourteen rheumatoid-arthritis patients (36 tendon reconstructions) were reviewed at an average of 54.1 months post surgery (range, 40 – 72). The average range of MCP-joint flexion subsequent to reconstruction was 66°. Following surgery, the extension lag at the metacarpophalangeal joint had been significantly improved from a preoperative mean of 38° (range, 25°–60°) to a postoperative mean of 16° (range, 0°–30°) (*p *< 0.05). (Table 2) (Fig. 2) We found good functional improvements for the patients participating in our study, following interpositional grafting using palmaris longus tendon in order to reconstruct extensor tendon defects for the study-participating rheumatoid-arthritis patients.

**Table 2:** Functional Results of Patients

Case	Extension Lag pre-op	Extension Lag post-op	Postoperative MCP joint flexion	Ability to Work (Percent)	F/U (mos)
1	28	9	85	100	51
2	45	25	66	100	53
3	53	28	55	100	47
4	29	13	73	100	41
5	30	18	78	100	69
6	45	20	65	100	68
7	60	24	40	100	44
8	50	30	65	100	40
9	35	15	57	100	46
10	25	5	53	100	66
11	42	16	63	75	58
12	36	18	60	100	72
13	27	8	76	100	42
14	25	0	85	100	60

MCP: metacarpalphalangeal

### Work status

Subjectively, all patients revealed that they were satisfied with the clinical results of their surgical procedure, and all had achieved a return to their previous occupations. One patient returned to light work (75 percent of the preinjury capacity) but had no difficulty with functions of daily living and his avocation at the time of the 58-month follow-up examination. Thirteen patients, however, continued the same occupation at 100 percent of the preinjury capacity including housekeeping.

### Complication

There were no serious postoperative complications. No patient lose MCP flexion due to the graft overtensionor MCP joint extension contracture with average of follow-up was 54.1 months (range, 40 to 72 months).

## Discussion

The extensor tendons and soft-tissue envelope are frequently compromised for RA patients. For patients suffering wrist level extensor tendon rupture associated with rheumatoid arthritis (RA), primary tendon repair is generally not feasible. This relates to the diffuse nature of the tendon damage that typically occurs in such patients, combined with fibrosis, atrophy, and retraction of the muscle, events that usually preclude effective tendon repair.

Several different methods have been previously used to repair finger extensor-tendon ruptures at wrist level for the rheumatoid-arthritis patient [3-8]. Primary repair involves the end-to-end suture of the two ends of the damaged tendon, and, as best we are aware, is generally not feasible for rheumatoid patients, as a significant length of tendon is typically damaged by the mechanical attrition and inflammatory process that occurs following tendon rupture. When multiple tendons are ruptured, the results of such surgical repair by any of these techniques are often unsatisfactory. The diffuse nature of the tendon damage, combined with fibrosis, atrophy, and retraction of the muscle, usually precludes repair. When rupture is diagnosed early, tendon grafting may be successful [9]. Some authors think that tendon grafting resulted in good correction of extension lag, but patients were dissatisfied with accompanying loss of digital flexion because of the long standing nature of the disease and decreased musculotendinous unit excursion, leading to loss of flexion following grafting [10]. Good results have been reported for tendon grafts, provided that the time from tendon rupture to surgery is short and muscle contracture is not allowed to become severe [9,11,12].

Interpositional grafting is able to be used as a surgical-repair technique for ruptured extensor tendons in order to overcome the problem of defects in the extensor mechanism where a portion of the relevant tendon has irreparably damaged and effectively lost. The tendon graft can be placed directly in between the ruptured extensor-tendon ends, or alternatively, re-routed subcutaneously in order to avoid the diseased tendon bed [5]. Interpositional tendon grafting using palmaris longus to repair extensor-tendon defects has previously been described as constituting a technique that can be effectively used for the repair of ruptured finger-extensor tendons [13]. The palmaris longus is the tendon of choice because it fulfils the requirements of length, diameter, and availability without producing a deformity. This choice of technique for tendon grafting features the advantage of the source of donor tendon being readily accessible in the same forearm.

The presence of this tendon should be determined before any grafting procedure. The tendon is reported to be present in one arm in 85% of people and in both arms in 70%. If the PL tendon absence occurred in our patients who need autogenous tendon graft to reconstruction, long extensors of toes were our second choice. The extensor of the third toe is probably easiest to remove and use. The method is to make multiple short transverse incisions over the tendon and remove it by elevating the skin proximal to each incision and dissecting to a more proximal level; then make another incision at this point and repeat the procedure. Extract the divided end of the tendon through each successive incision and remove it through the proximal incision.

On the basis of the results of the study, good functional outcomes can be achieved with end-to-end tendon-grafting technique using autogenous palmaris longus tendon graft. The mean extension lag of the metacarpophalangeal joint following tendon grafting for our study participants was 16.4°, a figure which was somewhat better than the 30° figure reported in 1987 by Bora et al. [9]. Further, we observed that metacarpophalangeal-joint flexion was improved for all patients subsequent to surgery.

Promoting tendon healing and avoiding joint adhesion are critical parts of the postoperative management of tendon reconstruction following tendon rupture. In our study, the Pulvertaft technique of weaving two tendons together was used, it provides a very-strong connection between the two grafted tendon ends, and a surgical repair technique that can then be loaded more quickly. The feature of this technique makes the "early active" type of rehabilitative protocol (we allowed the wrists active flexion within one month postoperatively in our series) feasible for patients having undergone such a surgical-repair technique.

Dynamic splinting following extensor tendon repair is becoming increasingly popular. The use of dynamic outrigger splints which allow active flexion and extension of the interphalangeal (IP) joints and active flexion but only passive extension of the MP joints. The dynamic splint combined with the tendon mobilization program provided the gliding necessary and was easy for the patient to comply with and understand [14]. Some studies relating to "early active" motion post surgical repair of ruptured extensor tendons have shown that patients who undergo early controlled, dynamic motion experienced improved damaged-hand function more rapidly than was the case for those more-immobilized patients, this shortening the overall total rehabilitation time required post such injury, and making dynamic motion treatment highly cost effective [15].

Reconstruction for multiple tendon ruptures is a salvage procedure that is often associated with extensor lag and impairment of overall hand function. For our study, the mean age at surgery for study participants was 47.3 (range, 32–66) years, the functional requirements of the injured hands of the patients participating in our study being highly "in demand" prospectively as regards these individuals' working lives. The average range of MCP-joint flexion and the extension lag at the metacarpophalangeal joint for our study participants was shown to be improved significantly following reconstruction using autogenous palmaris longus tendon grafting.

## Conclusion

In conclusion, multiple extensor-tendons reconstruction using autogenous palmaris longus tendon grafting for highly demand middle-aged rheumatoid arthritis patients is another viable option in order to achieve good clinical functional results post-operatively.

## Competing interests

The authors declare that they have no competing interests.

## Authors' contributions

PJC drafted the manuscript. PJC, HML and JTS participated in the design of the study. All authors conceived of the study, and participated in its design and coordination and helped to draft the manuscript. All authors read and approved the final manuscript.
